# Acceleration of Bone Healing by In Situ-Forming Dextran-Tyramine Conjugates Containing Basic Fibroblast Growth Factor in Mice

**DOI:** 10.7759/cureus.10085

**Published:** 2020-08-27

**Authors:** Shintaro Shoji, Kentaro Uchida, Wataru Saito, Hiroyuki Sekiguchi, Gen Inoue, Masayuki Miyagi, Akiyoshi Kuroda, Masashi Takaso

**Affiliations:** 1 Orthopaedic Surgery, Kitasato University School of Medicine, Sagamihara, JPN; 2 Orthopaedics, Shonan University, Chigasaki, JPN

**Keywords:** in situ-formed gel, dextran, basic fibroblast growth factor, fracture

## Abstract

An enzymatic crosslinking strategy using hydrogen peroxide (H_2_O_2_) and horseradish peroxidase (HRP) has been receiving increasing attention for use with in situ-formed hydrogels (IFHs). Several studies have reported the application of IFHs in cell delivery and tissue engineering. IFHs may also be ideal carrier materials for bone repair, although their potential as a carrier for basic fibroblast growth factor (bFGF) has yet to be evaluated. Here, we examined the effect of an IFH made of dextran (Dex)-tyramine (TA) conjugates (IFH-Dex-TA) containing bFGF in promoting bone formation in a fracture model in mice. Immediately following a fracture procedure, animals either received no treatment (control) or an injection of IFH-Dex-TA/phosphate-buffered saline (IFH-Dex-TA/PBS) or IFH-Dex-TA containing 1 μg bFGF (IFH-Dex-TA/bFGF) into the fracture site (n=10, each treatment). Fracture sites injected with IFH-Dex-TA/bFGF showed significantly greater bone volume, mineral content, and bone union than sites receiving no treatment or treated with IFH-Dex-TA/PBS alone (each n=10). This Dex-TA gel may be an effective drug delivery system for optimizing bFGF therapy.

## Introduction

About 5-10% of fractures result in delayed or poor non-union healing at the fracture site. These cases may lead to functional disability due to deformed healing or pseudoarthrosis [1]. Therefore, the use of bioactive materials that encourage the bone formation and healing may improve fracture healing.

One method that is used to increase the speed of fracture healing involves the local application of growth factors [2]. Methods that aim to promote bone formation via the sustained release of growth factors using various carriers have been reported. One growth factor known to be active at fracture-healing sites is basic fibroblast growth factor (bFGF). Fibroblast growth factors (FGFs) consist of a family of 23 structurally related polypeptides that play a critical role in angiogenesis and mesenchymal cell mitogenesis [3,4]. bFGF is expressed in periosteum during mesenchymal cell proliferation and chondrogenesis and promotes the growth of many types of cells, such as osteoblasts and chondrocytes [2,5-7]. Among FGF family members, the accumulation of bFGF is greatest in the bone matrix, and it is expressed in periosteum early in bone formation [5,8,9]. In several animal-model studies, locally applied recombinant human bFGF (rhbFGF) has shown osteogenic properties in the regeneration of bone fractures and defects, as well as osteoporotic bone [10-12]. Moreover, several clinical trials have recently reported that bFGF accelerates bone union following osteotomy and in tibial shaft fractures [2,7]. These properties indicate that bFGF is effective in promoting bone formation and is a growth factor with therapeutic potential in clinical settings. However, despite this osteogenic potential of bFGF, its efficiency diminishes rapidly following the diffusion in body fluid from bone defect sites [13]. Moreover, bFGF at high doses can produce adverse side effects, including thrombocytopenia, renal toxicity, and malignant cell activation [14,15]. Accordingly, the use of bFGF should ideally be restricted to a form where it is combined with a carrier to promote retention at wound sites. This in turn highlights the need for growth factor delivery carriers that provide the sustained release of bFGF at fracture sites [10-12,16].

Implantable carriers such as absorbable collagen sponge or hydroxyapatite have been used to aid fracture healing in clinical settings. However, these biomaterials require surgical incision for implantation, and the method is accordingly invasive [17]. In contrast, injectable materials have the advantage of being less invasive than implantable materials but, compared to implantable materials, generally diffuse only from the injection site [18]. Therefore, a material that is injectable and has the advantages of an implantable material may be an ideal candidate for a bFGF carrier. In this regard, attention has been recently focused on an enzymatic crosslinking strategy using hydrogen peroxide (H_2_O_2_) and horseradish peroxidase (HRP) for use with in situ-formed hydrogels (IFHs) made of natural polysaccharides, such as dextran (Dex), pullulan, and hyaluronic acid [19]. IFHs have suitable properties for biomedical applications, including good cytocompatibility, tunable reaction rate, and substrate specificity, and several studies have reported their use in cell delivery and tissue engineering for bone or cartilage repair [20-22]. IFHs may also be ideal carrier materials for bone repair, although their potential as a carrier for bFGF has yet to be examined.

Here, we examined the effect of an IFH made of Dex (IFH-Dex) containing bFGF for promoting osteogenesis in a fracture model in mice.

## Materials and methods

Synthesis of dextran-tyramine conjugates (Dex-TA)

Dextran-tyramine conjugates (Dex-TA) were synthesized by referring to previous reports [23]. Dextran was combined with PNC to form derivatives of p-nitrophenyl carbonate, which were treated with tyramine (TA) by aminolysis. Dextran produced by Meito Sangyo Co. (40 g, 471 mmol OH) (Meito Sangyo Co., Ltd., Nagoya, Japan) was dissolved in DMF (1,600 mL, containing LiCl 30.9 g) under nitrogen at 90 ˚C. After the dextran was dissolved, the mixture was allowed to cool and at 0 ˚C. PNC (23.8 g, 120 mmol) and pyridine (9.2 ml) were combined with the solution under stirring. The feeding molar ratio of PNC to hydroxyl groups with dextran was about 0.25. The reaction was allowed to continue overnight. Dextran activated with p-nitrophenyl carbonate groups (denoted as Dex-PNC) was then precipitated in cold ethanol (2,000 ml), followed by filtering and careful washing with ethanol and diethyl ether, and drying in a vacuum oven.

Subsequently, Dex-PNC was dissolved in 740 mL of DMF, and TA (9.1 g, 65 mmol) was added under nitrogen. The reaction was continued for three hours at room temperature. The product was then precipitated in cooled ethanol (800 ml), filtered, and washed carefully with diethyl ether and ethanol. The Dex-TA conjugates were purified further using ultrafiltration against deionized water and isolation following lyophilization. 1H NMR was used to establish the composition of the Dex-TA conjugates. The degree of substitution (DS) (1H NMR) was 12. 1H NMR (D2O): d 2.60 and 2.88 (m, -CH2-CH2-), 3.20-3.84 (m, dextran glucosidic protons), 4.84 (s, dextran anomeric proton), 6.72 and 7.01 (m, TA aromatic protons). DS, defined as the number of substituents/100 anhydroglucosidic rings (AHG rings) in dextran, was evaluated using 1H NMR by comparison of signal integrals at d 5.0 and d 6.5-7.5 for Dex-TA, in reference to the previous method [23].

Preparation of IFH-Dex-TA

IFH-Dex-TA was prepared by cross-linking Dex-TA polymer in the presence of HRP as the catalyzing enzyme, and H_2_O_2_ in 10 mM phosphate-buffered saline (PBS; pH 7.4). Briefly, Dex-TA polymer solution (final concentration: 2% w/v) was combined with 0.8 units/mL HRP solution (final concentration: 0.8 units/mL) containing 1 µg bFGF (IFH-Dex-TA/bFGF) or PBS (IFH-Dex-TA/PBS) and H_2_O_2_ solution (final concentration: 4 mM).

Mouse fracture model

The femur fracture model was produced in C57BL/6J mice aged nine weeks [24]. The mice were maintained at Nippon Charles River Laboratories (Kanagawa, Japan) in a semi-barrier system with controlled temperature (23 ±2 °C), humidity (55 ±10%) and lighting (12-h light/dark cycle), and received standard rodent chow (CRF-1; Oriental Yeast, Tokyo, Japan). The fracture model was generated by producing a 10-mm incision on the lateral side of the left thigh under sterile conditions. The left patella was medially dislocated by producing a 4-mm lateral parapatellar incision. Following the drilling of a 0.5-mm hole in the intercondylar notch, a stainless steel needle (0.5-mm diameter) was retrogradely inserted into the intramedullary canal. The osteotomy was conducted using a wire saw of 0.22-mm diameter via a small lateral approach, and insertion of a stainless steel needle into the intramedullary canal was used for stabilization. Immediately following the fracture, the animals either received no treatment (control) or received an injection of IFH-Dex-TA/PBS or IFH-Dex-TA/bFGF in the fracture site (n=8, each treatment). All animal experiments were conducted in accordance with the guidelines of the Animal Ethics Committee, Kitasato University (approval number: 2019-127).

Determination of new bone volume and bone mineral content

All mice were sacrificed four weeks after treatment. Femurs along with the surrounding muscle were removed and fixed in 4% paraformaldehyde for 48 hours at 4 °C. The femurs were moved into PBS and imaged on a micro-focus X-ray CT system (inspeXio SMX-90CT; Shimadzu, Tokyo, Japan) using a 90 kV acceleration voltage, 110 mA current, 20 lm/pixel voxel size, and 1,024 × 1,024 matrix size. Using the micro-CT images of the whole femur, newly developed bone volume and bone mineral content were quantified in a 10-mm region of interest centered on the fracture site (500 slices) chosen at the shaft of the femur for each animal using a 3-dimensional (3D) image analysis software application (Tri-3D-Bon; Ratoc System Engineering, Tokyo, Japan), as reported previously. Regions of new bone were determined with a threshold density of 300 mg/cm^3^ [18,24].

Histology

The bone formation mechanism induced by IFH-Dex-TA/bFGF was assessed by excising femurs from the control and treated animals four weeks after the production of fractures. They were dematerialized in a solution of 20% ethylenediaminetetraacetic acid (EDTA) for four weeks. Residual tissue was embedded in paraffin, and 3-µm coronal sections were cut along the long axis of each femur. These sections were processed by hematoxylin and eosin (HE) staining for morphological evaluation.

Sustained in vitro release of bFGF

To assess the sustained release of bFGF from IFH-Dex-TA, H_2_O_2_ solution containing Dex-TA and HRP solution containing 1 µg bFGF were added to a 0.5-mL plastic microcentrifuge tube. After curing IFH-Dex, 200 μl of PBS was added to the tube. To determine the release of bFGF from IFH-Dex-TA, bFGF-loaded microtubes were incubated in 200 μl of PBS for one, four, eight, 24, 48, and 72 hours. The supernatant was collected and kept at -30 °C until assay. The concentration of bFGF was estimated using a commercial ELISA kit (R&D Systems, Minneapolis, MN).

## Results

Dex gel containing bFGF induced callus formation in vivo

**Figure 1:**
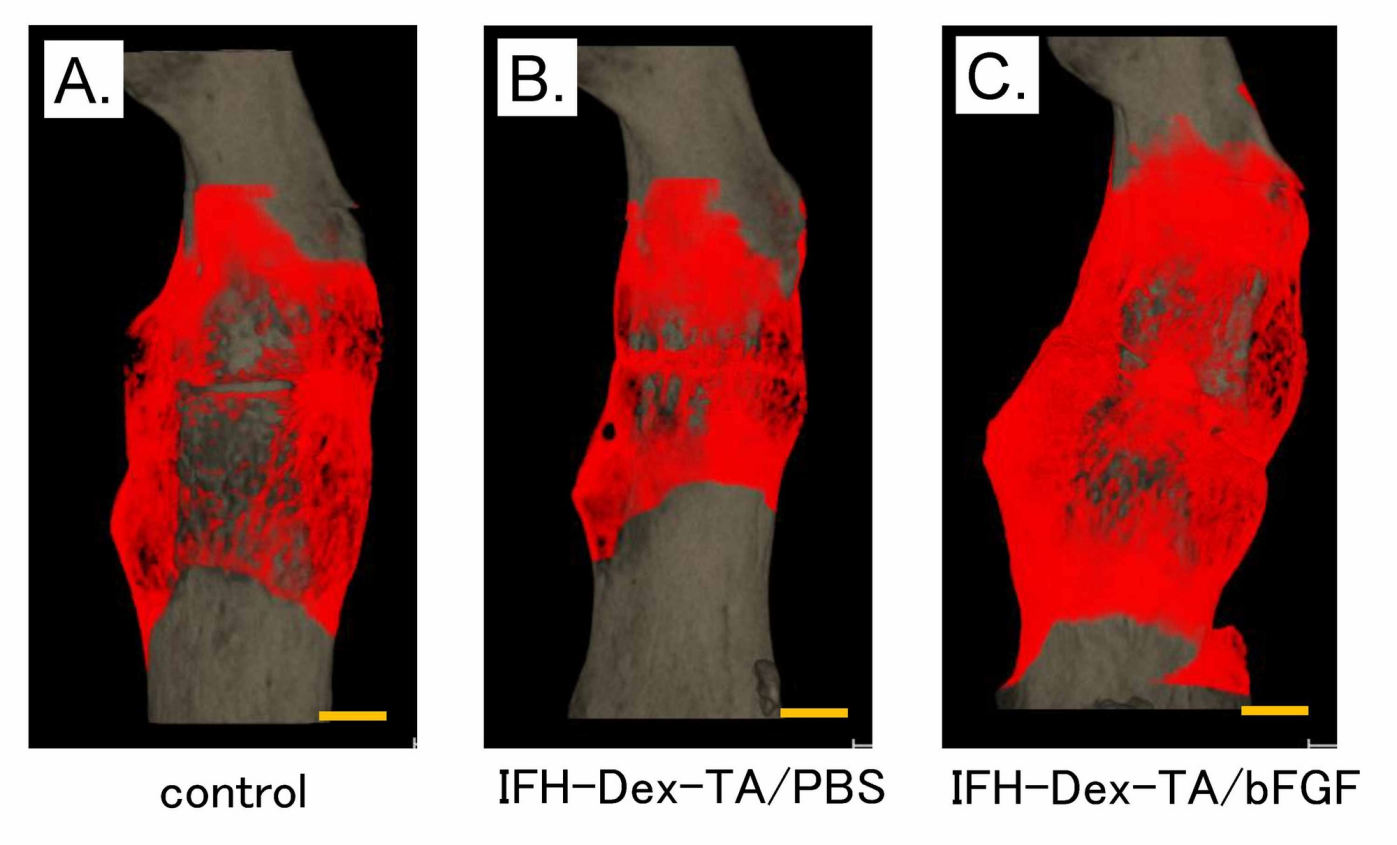
Representative 3D micro-CT image of femurs following injection of in situ-formed hydrogel made of dextran (IFH-Dex-TA) loaded with bFGF 3D micro-CT images of fractured femurs from (A) control, (B) IFH-Dex-TA/PBS-, and (C) IFH-Dex-TA/bFGF-treated groups after four weeks of recovery. Red: new bone formation; gray: existing bone CT: computed tomography; IFH: in situ-formed hydrogels; Dex: dextran; TA: tyramine; PBS: phosphate-buffered saline; bFGF: basic fibroblast growth factor

We evaluated callus formation in the fractured femurs following treatment with IFH-Dex containing bFGF using micro-CT image analysis at four weeks post-treatment (Figure 1).

**Figure 2:**
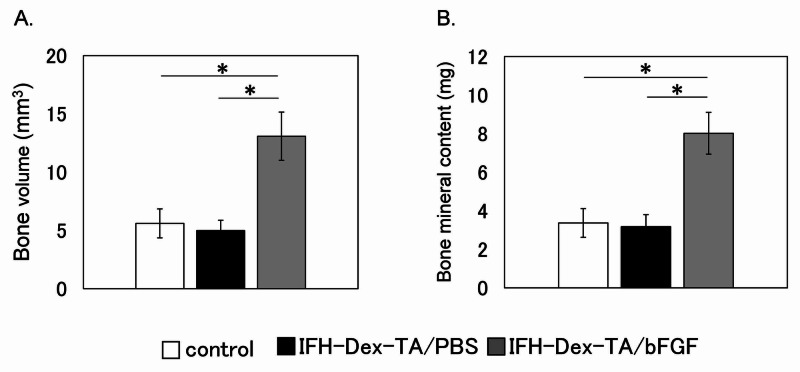
Quantification of callus area and bone mineral content at the fracture site four weeks following the creation of the fracture Analysis of (A) bone volume (mm^3^) and (B) bone mineral content (mg) in calluses from control (white bars), IFH-Dex-TA/PBS- (black bars), and IFH-Dex-TA/bFGF-treated (gray bars) groups. Data are shown as the mean ± standard error (SE) (n=8) *p: <0.05 versus the control group IFH: in situ-formed hydrogels; Dex: dextran; TA: tyramine; PBS: phosphate-buffered saline; bFGF: basic fibroblast growth factor

Compared to sites that received no treatment (control) or were treated with IFH-Dex alone, fracture sites injected with IFH-Dex-TA/bFGF showed significantly greater bone volume and bone mineral content (Figure 2) (p<0.05). In contrast, these variables were comparable between the IFH-Dex-TA and control groups.

Histomorphometric findings

**Figure 3:**
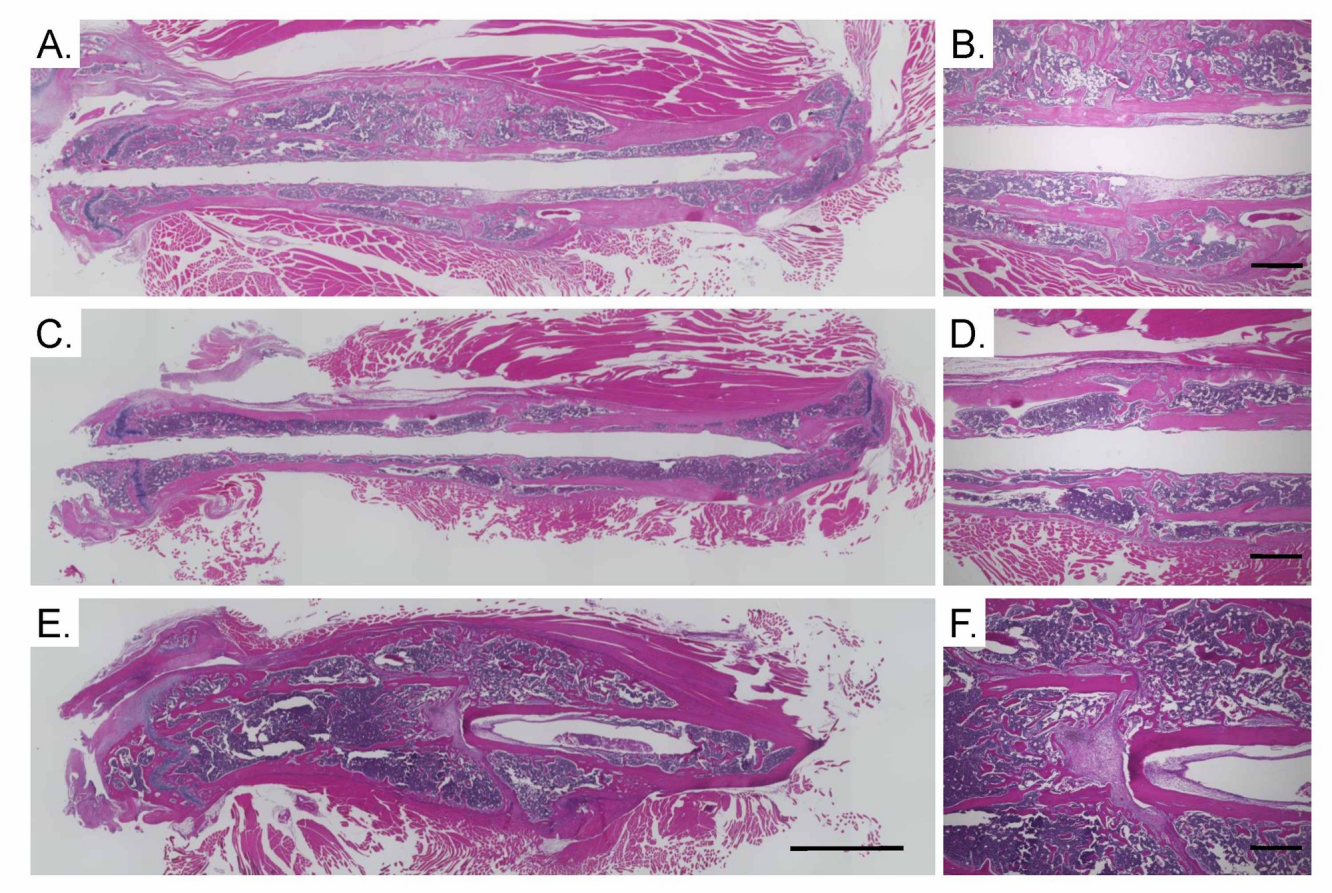
Hematoxylin and eosin (HE) staining of the femur and surrounding muscle (A–B) control, (C–D) IFH-Dex-TA/PBS, and (E–F) IFH-Dex-TA/bFGF. Scale bars indicate 2 mm (A, C, E) or 0.5 mm (B, D, F) IFH: in situ-formed hydrogels; Dex: dextran; TA: tyramine; PBS: phosphate-buffered saline; bFGF: basic fibroblast growth factor

To evaluate bone union, we conducted a histological examination of the fracture site four weeks post-fracture. The IFH-Dex-TA/bFGF-treated group exhibited large calluses at the fracture site, and the fracture site was bridged by newly formed bone (Figure 3). In contrast, in the IFH-Dex and control groups, small calluses were observed at the fracture site (Figure 3).

Sustained release of bFGF from IFH-Dex-TA in vitro

**Figure 4:**
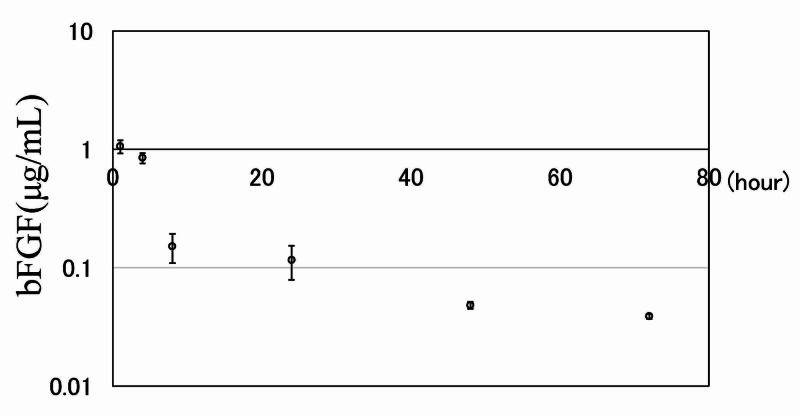
Sustained release of bFGF from IFH-Dex-TA gel in vitro bFGF concentration in PBS at different time points. Results are presented as mean ± standard error (SE) (n=5) IFH: in situ-formed hydrogels; Dex: dextran; TA: tyramine; PBS: phosphate-buffered saline; bFGF: basic fibroblast growth factor

The in vitro profile of bFGF release from IFH-Dex-TA is shown in Figure 4. bFGF release from Dex-TA gel occurred with an initial burst in the first four hours followed by a gentler release pattern after eight hours. Thereafter, the sustained release rate was moderate, with 37% of the administered dose of bFGF gradually released across 72 hours.

## Discussion

Previous studies have reported that bFGF combined with carriers having various forms, including powders, sheets, sponges, gels, has an effect on the bone to promote bone formation [2,10-12,16,18,25]. Dextran protects bFGF from acid and heat inactivation and proteolysis, and its protective effect is stronger than that of heparin, a known bFGF stabilizer [26]. Dextran gel is gradually released from bFGF and promotes angiogenesis [27]. In our present study, 1 μg bFGF with in situ-formed hydrogels composed of Dex-TA induced accelerated bone formation at the fracture site in mice. We previously showed that 1 μg bFGF combined with artificial collagen gel failed to accelerate bone formation in a mice fracture model [18]. In addition, even when 10 μg bFGF combined with collagen powder was administered to the fracture site, bone formation was not accelerated [25]. Accordingly, this IFH-Dex-TA gel may be useful as a carrier for bFGF to accelerate bone formation.

When using various substances as carriers for growth factors, it is important that the growth factors be released slowly. bFGF is reported to have a growth-promoting effect on undifferentiated mesenchymal cells at an early stage in the process of fracture healing [11]. When administered directly into the body, it diffuses rapidly. However, because it is considered to produce its activity by affecting the initial stage of the bone union process [11,13], it is important that the release occurs locally in order to minimize or prevent diffusion. The IFH-Dex-TA gel containing bFGF provided a large and sustained release of bFGF in the first four hours after injection. The amount released thereafter decreased, but the bFGF concentration in the PBS solution after 72 hours was 38.7 ng/mL. In this regard, bFGF was reported to show proliferative activity on periosteal cells at a concentration of 1 ng/mL in vitro [28]. The proliferation of periosteal cells occurred from day one to three after the creation of a fracture in a fracture model in mice [29,30]. Accordingly, we speculate that bFGF-containing IFH-Dex-TA gel could release a sufficient amount of bFGF to exert a cell-growth-promoting effect during fracture healing.

There were two main limitations to this study. The release kinetics of bFGF in vivo remain unclear. The usage of fluorescently-labeled bFGF was needed to reveal the release kinetics. Moreover. extrapolating the results obtained from small animal models directly to man may not be clinically relevant. We recommend further investigation using large animals.

## Conclusions

We examined the osteogenesis-promoting ability of Dex gel containing bFGF in a fracture model in mice. Fracture sites injected with Dex/bFGF showed significantly greater bone volume and bone mineral content than sites receiving no treatment or treated with Dex gel alone. The use of this Dex gel as a drug delivery system may be effective for optimizing bFGF therapy.
